# Technological advances in axonal regeneration and structural plasticity: optogenetics, single-cell omics, and tissue clearing

**DOI:** 10.3389/fncel.2026.1857896

**Published:** 2026-07-27

**Authors:** Xiaoqiu Shu, Jiawen Zhang, Zhenwei Qin

**Affiliations:** 1 Deqing People’s Hospital, Deqing Campus, Sir Run Run Shaw Hospital, School of Medicine, Zhejiang University, Huzhou, China; 2 Sir Run Run Shaw Hospital, School of Medicine, Zhejiang University, Hangzhou, China; 3 Eye Center, The Second Affiliated Hospital, School of Medicine, Zhejiang University, Hangzhou, China

**Keywords:** 3D imaging, axon regeneration, optic nerve injury, optogenetics, single-cell RNA sequencing, spinal cord injury, tissue clearing

## Abstract

Modern techniques have provided novel insights into the molecular and structural characteristics of neurons and glial cells in the central nervous system (CNS). Over the past decade, advancements in optogenetics, single-cell RNA sequencing, and tissue clearing have enabled unprecedented resolution, sensitivity, and throughput, driving progress in CNS repair. In this review, we focus on how these technologies have illuminated the dynamic structural and functional behaviors of axons, from degeneration to regeneration, following spinal cord and optic nerve injuries. Optogenetics offers precise spatiotemporal control of neuronal activity, revealing how targeted stimulation influences axonal growth and circuit reorganization. Single-cell omics uncovers the heterogeneity of axonal regenerative capacity among neuronal subtypes and identifies novel regeneration-associated genes (RAGs) that orchestrate axonal structural remodeling. Tissue clearing and 3D imaging allow direct visualization of axonal trajectories, growth cone morphology, and pathfinding errors in intact tissue, overcoming limitations of conventional histology. Together, these approaches have provided important insights into axonal dynamics and have opened new avenues for promoting functional recovery after CNS injury.

## 1 Introduction

Most neurons in the adult mammalian central nervous system (CNS) exhibit limited spontaneous axon regeneration after injury, although regenerative capacity varies across neuronal subtypes, injury models, and experimental conditions. Damaged axons rarely regrow spontaneously to their original targets, and adult stem cells contribute only modestly to neuronal replacement (Denoth-Lippuner and Jessberger, 2021; Tuszynski and Steward, 2012; Varadarajan et al., 2022). Thus, CNS injury can result in persistent structural damage and long-term functional deficits, although the extent of anatomical and functional recovery varies across injury models and experimental contexts. To date, two valuable models for studying mammalian CNS regeneration have been the spinal cord injury (SCI) and optic nerve crush (ONC) models (Alizadeh et al., 2019; Fischer et al., 2017).

SCI and ONC represent two complementary and well-established mammalian CNS injury models for investigating axonal regeneration and structural plasticity. SCI provides a clinically relevant model for analyzing long-distance axon regrowth, lesion microenvironment remodeling, glial responses, relay circuit formation, and motor recovery. In contrast, ONC offers a well-defined and experimentally accessible CNS model in which retinal ganglion cells (RGCs), optic nerve axons, and central visual targets can be examined for neuronal survival, subtype-specific regenerative competence, axon guidance, target reinnervation, and functional visual recovery. Together, SCI and ONC provide complementary platforms for applying optogenetics, single-cell omics, and tissue clearing/3D imaging to connect functional manipulation, molecular profiling, and structural visualization of CNS axon repair (Varadarajan et al., 2022).

Although tremendous effort has been devoted to the characterization of cellular and molecular mechanisms underlying regeneration failure in both SCI and ONC models, a better and more comprehensive understanding of regeneration and degeneration is still lacking. To date, promoting clinically meaningful and functionally integrated regeneration of human CNS neurons remains an unresolved therapeutic challenge.

Over the past decade, interdisciplinary collaborations have catalyzed the emergence of innovative technologies in neuroscience. Technological and analytical advances, spanning molecular and cellular analyses to systems-level behavioral studies, have enabled researchers to address previously inaccessible scientific questions and have propelled neuroscience forward. The applications of emerging technologies, such as single-cell sequencing (scRNA-seq) (La Manno et al., 2018; Stuart and Satija, 2019; Tang et al., 2009), optogenetic methods in neural manipulation (Deisseroth, 2015; Packer et al., 2013; Zhang Z. et al., 2018), tissue clearing and 3D imaging methods (Chung and Deisseroth, 2013; Chung et al., 2013; Muntifering et al., 2018; Vieites-Prado and Renier, 2021) have significantly broadened the scope of neuroscience research. These advances have provided important tools for studying neurobiology and axon regeneration. For example, the application of single-cell RNA sequencing technology has facilitated the identification of distinct cell subtypes and various signaling pathways that regulate regeneration in both spinal and visual systems models (Li et al., 2020; Li T. et al., 2023; Menon et al., 2019; Rheaume et al., 2018). Importantly, a variety of key mechanistic discoveries have been made that point towards new directions for the field of CNS regeneration.

Here, we primarily introduce three novel technologies: optogenetics, single-cell RNA sequencing, and the tissue clearing method. We summarize their use as powerful tools for elucidating both extrinsic and intrinsic mechanisms underlying axon degeneration and regeneration after CNS injury. Importantly, we emphasize how these technologies have advanced our understanding of axonal structural dynamics, including the initiation of growth cones, the remodeling of axonal trajectories, and the intrinsic heterogeneity of regenerative capacity among different neuronal subtypes. By integrating findings from optogenetic manipulation, single-cell transcriptomics, and 3D tissue imaging, this review provides a comprehensive update on the functional and structural dynamics of axons in the injured CNS, with a particular focus on spinal cord and optic nerve models.

Conceptually, these technologies address complementary levels of axon biology. Single-cell omics identifies cell types, molecular programs, and regeneration-associated genes that define the intrinsic regenerative competence of injured neurons. Optogenetics allows functional testing of how defined neuronal populations and activity patterns influence axonal sprouting, circuit remodeling, and recovery. Tissue clearing and 3D imaging provide structural validation by mapping axonal trajectories, growth cone morphology, and pathfinding errors in intact tissue. Importantly, anatomical axon regrowth does not necessarily indicate accurate target reinnervation or functional recovery, highlighting the need to evaluate molecular, structural, and functional outcomes together. Therefore, we use these three approaches as an integrated framework to connect molecular mechanisms, functional circuit testing, and structural visualization of axonal regeneration.

## 2 Optogenetic regeneration

Optogenetics enables selective manipulation of neuronal activity through genetically encoded light-sensitive proteins. By allowing defined neuronal populations to be activated or inhibited with high temporal precision, optogenetics provides a powerful approach for examining how neuronal activity regulates axonal growth, sprouting, and circuit remodeling after CNS injury (Mateus et al., 2024; Reshetnikov et al., 2022). Although microbial opsins such as ChR2 established the foundation for optical control of neuronal excitability, the relevance of optogenetics to axonal regeneration lies primarily in its ability to test causal relationships between activity patterns and injury-induced structural plasticity. Compared with electrical or pharmacological stimulation, optogenetic approaches provide greater cell-type specificity and temporal control, enabling more precise interrogation of activity-dependent regeneration mechanisms *in vitro* and *in vivo*. Given that applications of optogenetics to CNS axon regeneration remain comparatively limited, available studies are discussed with an emphasis on how optogenetic manipulation has been used to probe axonal sprouting, regeneration, and circuit repair after injury.

### 2.1 Optogenetics in spinal cord injury

Spinal cord injury (SCI) is a traumatic event caused by falls, traffic accidents, and other incidents. It results in severe neurological impairment, paralysis, and places a heavy burden on both the families and society of patients (GBD Spinal Cord Injuries Collaborators, 2023). Moreover, SCI exhibits a substantial mortality and disability rate (Simpson et al., 2012). To date, due to the difficulty of nerve injury repair and technical limitations, the clinical efficacy of spinal cord injury treatment remains inadequate. In contrast to PNS, adult mammalian CNS neurons, including spinal motor neurons, exhibit limited regenerative capacity. The limited intrinsic regenerative capacity and unfavorable extrinsic environment hinder the regeneration of CNS axons.

Clinically, electrical stimulation has broad potential for directly activating spinal cord neurons, but it also presents significant limitations. The electrical stimulation excites all nearby cells, leading to rapid muscle fatigue without specifically targeting any beneficial cell type. Furthermore, the use of electrical stimulation necessitates a long-term implant, thereby substantially augmenting the potential risks for patients (Li Y. et al., 2022). Compared to electrical stimulation, optogenetics selectively activates specific channels or molecules in unique neural subsets. For example, ChR2 can be expressed in spinal interneurons and motor neurons by viral transduction at the C3–C6 level of the spinal cord, providing a cell-type-specific approach for testing activity-dependent mechanisms of axonal regeneration (Alilain et al., 2008; Ramer et al., 2014).

Calcium-dependent signaling pathways play important roles in neural regeneration by promoting growth cone formation (Ghosh-Roy et al., 2010; Hendricks and Shi, 2014; Kamber et al., 2009; Watkins et al., 2013). Mechanistically, optogenetic stimulation may influence axonal regeneration through several activity-dependent pathways. Activation of ChR2-expressing spinal neurons induces rapid membrane depolarization and Ca^2 +^ influx, linking light stimulation to intracellular calcium signaling (Nagel et al., 2003). Local calcium dynamics at injured axonal tips can determine whether a cut axon forms a retraction bulb or a competent growth cone, suggesting that optogenetically induced Ca^2 +^ signaling may affect early growth cone conversion after injury (Kamber et al., 2009). In addition, Ca^2 +^/cAMP signaling and intrinsic growth pathways such as PI3K-Akt-mTOR are known to regulate axonal regeneration and regenerative competence (Ghosh-Roy et al., 2010; Park et al., 2008; Watkins et al., 2013). More recent SCI work further suggests that transcranial optogenetic stimulation promotes corticospinal tract axon regeneration through activation of the JAK2/STAT3 pathway, providing direct evidence that optogenetic stimulation can engage pro-regenerative molecular programs after SCI (Ma et al., 2025). Therefore, optogenetics should not only be viewed as a functional tool for activating spinal circuits, but also as a mechanistic approach for testing how defined patterns of neuronal activity regulate growth cone dynamics, axonal sprouting, and circuit rewiring after SCI.

Optogenetic technology is gaining recognition in studies of SCI. It is already being tested in animal models and clinical trials to restore spinal cord function. Recent studies have used optogenetics to investigate spinal cord circuitry involved in rebuilding limb movement (Hägglund et al., 2010; Hägglund et al., 2013). In order to facilitate clinical treatment, scientists have also developed humanized optogenetic devices for potential applications in nerve repair. For instance, Park SI embedded LED light sources in flexible materials, which can be sutured and attached to different spinal repair parts, achieving remote control of light stimulation with wireless devices (Park et al., 2015). Additionally, fusion expression of proteins can co-express opsins and luciferase, eliminating the need for a separate operation to implant an optical fiber for light delivery. This enables precise regulation of neuronal activity *in vivo* through bioluminescent luminescence, making optogenetic technology highly promising for spinal cord regeneration (Berglund et al., 2016). Together, these optogenetic studies reveal that light-controlled neuronal activity can dynamically influence axonal sprouting and growth cone behavior, providing a powerful tool to probe the structural plasticity of injured spinal axons.

**FIGURE 1:**
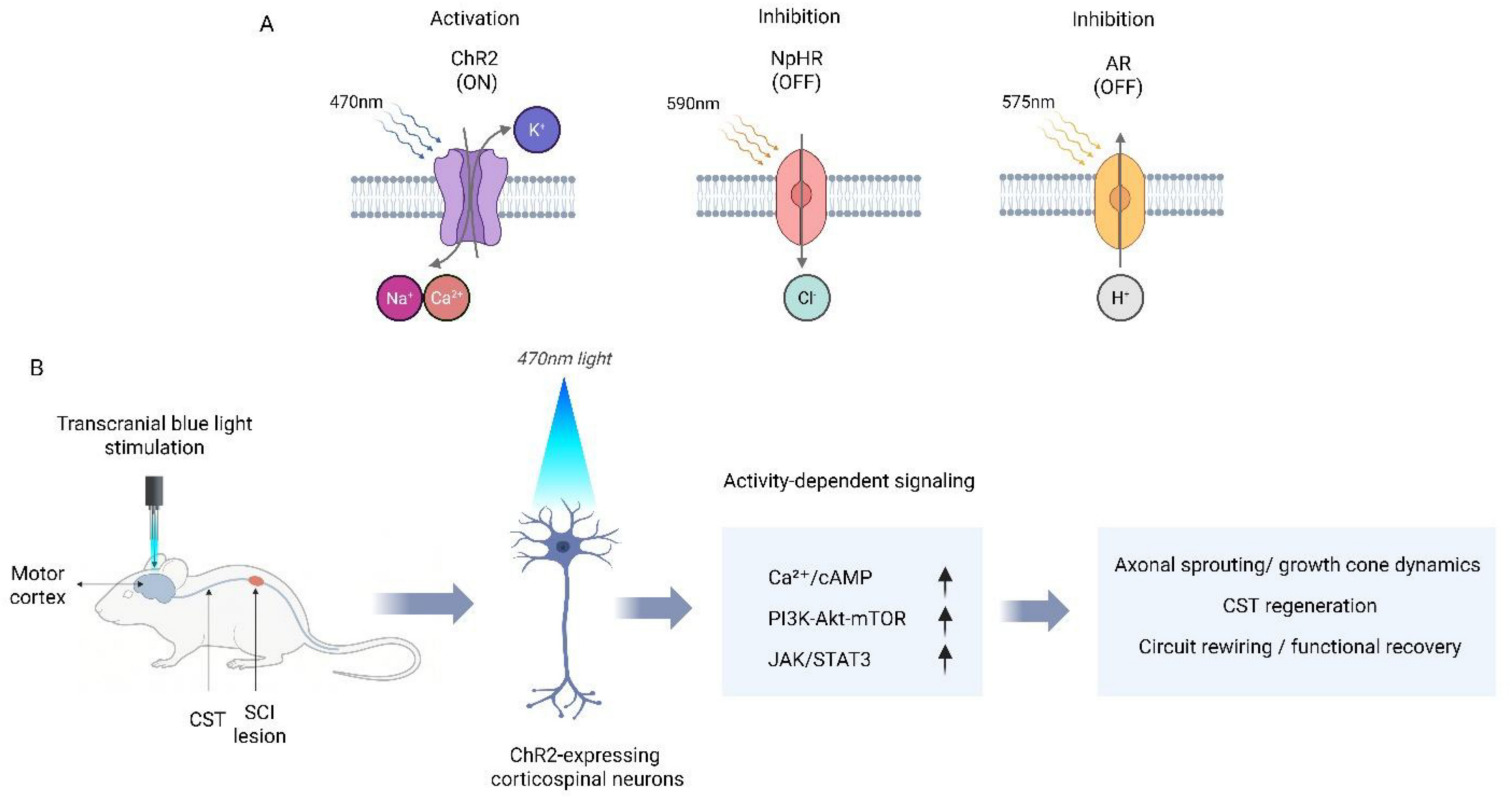
Optogenetic principles and activity-dependent axonal regeneration after spinal cord injury. **(A)** Neurons can be genetically targeted to express the excitatory opsin Channelrhodopsin-2 (ChR2) or the inhibitory opsins Halorhodopsin (NpHR) and Archaerhodopsin (AR). Activation of ChR2 with ∼470 nm blue light opens cation channels, leading to depolarization and neuronal excitation. In contrast, illumination of NpHR with ∼580 nm yellow light, or AR with ∼575 nm green light, causes chloride influx or proton efflux, respectively, resulting in hyperpolarization and neuronal inhibition. This allows precise, bidirectional control of neuronal activity in time and space. **(B)** Application of optogenetic stimulation to spinal cord injury-associated axonal regeneration. Transcranial blue-light stimulation can activate ChR2-expressing corticospinal neurons in the motor cortex. This activity-dependent manipulation may engage pro-regenerative signaling pathways, including Ca^2 +^/cAMP, PI3K-Akt-mTOR, and JAK2/STAT3 signaling, thereby promoting axonal sprouting, growth cone dynamics, corticospinal tract (CST) regeneration, circuit rewiring, and functional recovery after spinal cord injury.

Overall, optogenetics has substantially advanced axon regeneration research by shifting the field from correlative observations of neuronal activity to causal testing of defined neuronal populations, circuits, and stimulation patterns (Ma et al., 2025). However, several practical and translational barriers remain, including limited light penetration in deep CNS tissues, the need for invasive or implanted light-delivery devices, phototoxicity, altered membrane physiology or toxicity related to long-term opsin overexpression, and challenges associated with viral delivery, such as AAV tropism, transduction efficiency, tissue coverage, payload limitations, and immune responses to viral capsids or microbial opsins (McClements et al., 2020; Shen et al., 2020). These limitations are particularly important for translation because stimulation parameters, light intensity, opsin expression levels, delivery routes, and long-term safety have not yet been standardized across injury models. Therefore, optogenetics is most informative when combined with molecular profiling, structural imaging, and functional behavioral assays to determine whether activity-dependent axon growth leads to accurate target reinnervation and meaningful circuit repair (Figure 1).

### 2.2 Optogenetic vision restoration

For vision restoration, optogenetic therapy uses light-sensitive opsins to confer photosensitivity to surviving retinal neurons after photoreceptor degeneration. In this context, the major focus is how different opsins can be used to restore light responsiveness in the degenerated retina. Commonly used or engineered opsins, including ChR2, ChrimsonR, ChronosFP, melanopsin-based molecules, and mGluR6-based chimeras, differ in light sensitivity, kinetics, and target-cell suitability, which influence their potential use in retinal degeneration therapy (De Silva and Moore, 2022; Prosseda et al., 2022).

Efficient retinal delivery remains important for optogenetic vision restoration. AAV vectors are commonly used for retinal gene delivery because of their relative safety and established use in ocular gene therapy, and engineered AAV serotypes may further improve target-cell specificity and transduction efficiency (Balaggan and Ali, 2012; Puppo et al., 2014).

**FIGURE 2:**
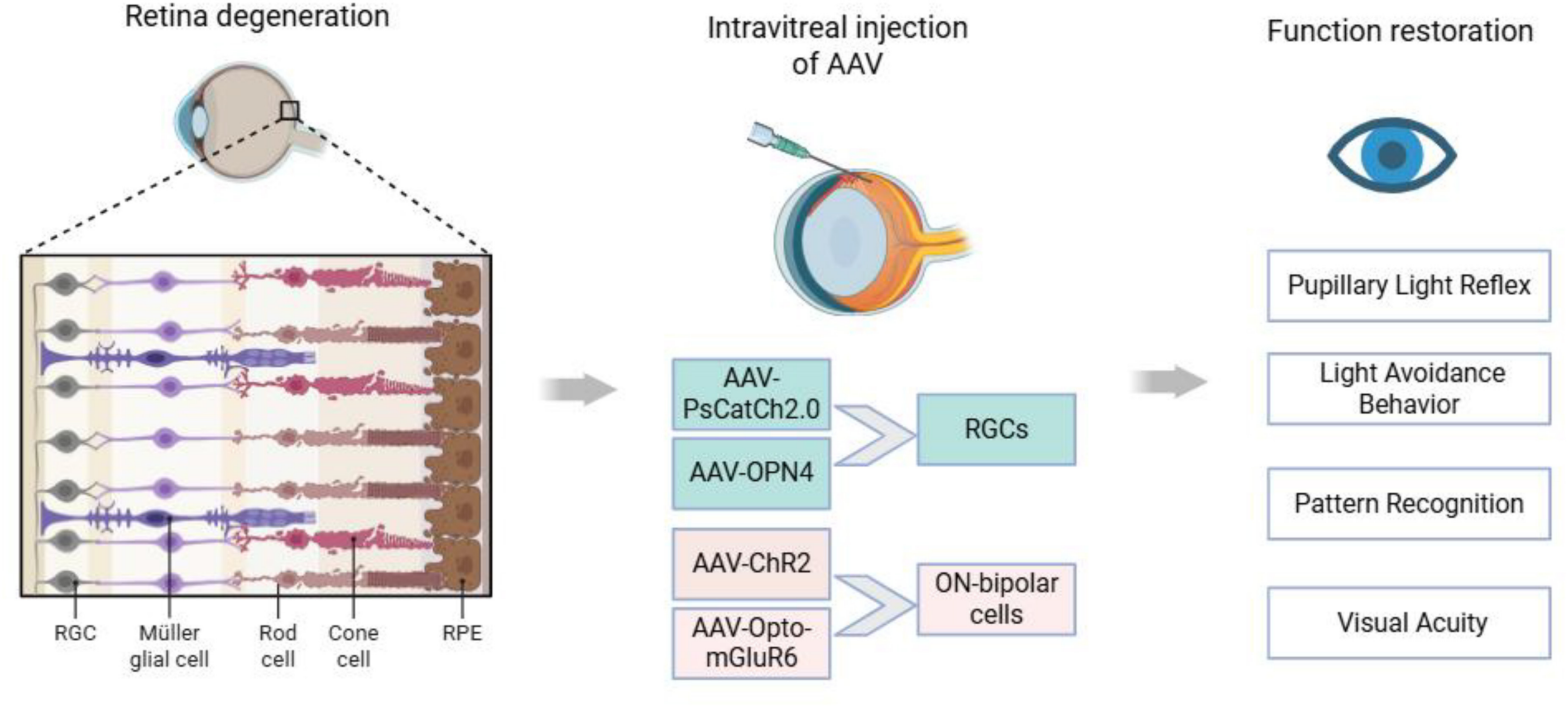
AAV-mediated optogenetic gene therapy restoring visual function. Intravitreal injection of AAV vectors delivers light-sensitive proteins to retinal cells, enabling the restoration of visual behaviors such as the pupillary light reflex and pattern recognition in blind mice.

Retinal degeneration primarily involves the loss of photoreceptors, which play a crucial role in facilitating high acuity central vision and color perception. Globally, retinal degenerations cause severe visual impairment, leading to eventual blindness and are estimated to affect millions of individuals (Hanany et al., 2020; Heath Jeffery et al., 2021). Several opsin molecules have been applied in the treatment of ophthalmic diseases (Barkana and Dorairaj, 2015; Sahel and Roska, 2013). Recently, a new photosensitive protein called PsCatCh2.0 was obtained through molecular engineering from PsChR. It has high sensitivity and fast kinetic properties, responding rapidly to light with a time resolution of at least 32 Hz. When PsCatCh2.0 is expressed in the retinal ganglion cells (RGCs) of rd1 mice, a model for retinal degeneration, it effectively improves their visual acuity to approximately 0.3 of human vision under safe light intensity (Chen et al., 2022). Ectopic expression of OPN4, a GPCR that activates cation channels in the cell membrane of ipRGCs, through AAV carrier transfection in RGCs of rd1 mice resulted in restoration of the pupil light reflex, behavioral light avoidance, and basic image recognition. Furthermore, OPN4-induced cells exhibited higher photosensitivity and slower but more sustained responses than ChR2-transduced RGCs (Hulliger et al., 2020; Kralik et al., 2022; Lu et al., 2016). Additionally, ChR2 expression in bipolar cells has been achieved through a recombinant adeno-associated viral vector (rAAV), resulting in ChR2-driven electrophysiological responses in retinal ganglion cells and significant improvement in visual function in the rd1 mice model (Doroudchi et al., 2011). A recent encouraging result suggests that Opto-mGluR6, OPN1MW-mGluR6, and two new melanopsin-mGluR6 chimeras may restore retinal vision in rd1 mice to levels similar to those of wild-type mice. This indicates the potential for restoring higher quality vision through targeted optogenetic gene therapies in human patients (Kralik et al., 2022; Figure 2). These findings underscore how optogenetic manipulation of surviving retinal ganglion cells can drive axonal remodeling and reconnect central visual targets, offering a blueprint for structure-guided repair.

### 2.3 Optogenetic therapy in the clinic

The retina serves as the primary target for optogenetic therapy in clinical settings. For patients with advanced retinal degeneration, optogenetic therapy aims to restore visual responses by delivering genes encoding light-sensitive proteins to surviving inner retinal neurons after photoreceptor loss. Current clinical approaches mainly target surviving RGCs as a broad inner retinal population, whereas other strategies target ON-bipolar cells to preserve more upstream retinal processing. It is reported that optogenetic application for vision recovery has led to at least four ongoing clinical trials (De Silva and Moore, 2022; Prosseda et al., 2022). All four retinal degeneration-targeting candidates are currently in phase 1/2 or phase 2 clinical trials for retinitis pigmentosa.

These clinical strategies are promising, but their translational interpretation depends on the target cell being manipulated. RGC-directed approaches directly activate retinal output neurons but bypass upstream retinal processing, whereas bipolar-cell-targeted strategies may better preserve retinal circuit computation but require efficient and cell-specific gene delivery.

GenSight Biologics developed GS030-DP (NCT03326336), which delivers the light-sensitive ChrimsonR-tdTomato gene to RGCs by intravitreal injection of an AAV2.7m8 capsid variant. Allergan’s RST-001 (NCT02556736) delivers an AAV vector encoding ChR2 to RGCs (Prosseda et al., 2022; Sahel et al., 2021). Nanoscope Therapeutics reported preliminary data from 11 patients with advanced retinitis pigmentosa who received two dose levels of the multi-characteristic opsin vMCO-010 (NCT04945772) through intravitreal rAAV2 delivery. The vMCO-010 candidate has been granted orphan drug designation in Europe and the United States. Furthermore, Bionic Sight developed BS01 (NCT04278131), which uses intravitreal delivery of a recombinant AAV2 vector expressing ChronosFP in RGCs during a phase 1/2 clinical trial for patients with advanced retinitis pigmentosa (De Silva and Moore, 2022; Prosseda et al., 2022). Despite these advances, clinical translation of retinal optogenetic therapy still faces several challenges, including relatively low light sensitivity compared with native photoreceptors, the possible need for external light-amplifying devices, variable AAV tropism and transduction efficiency, immune responses to viral vectors or microbial opsins, and uncertainty regarding long-term safety and durability. Future translational studies should systematically compare opsin properties, delivery routes, stimulation devices, and long-term functional outcomes.

While optogenetics provides a powerful approach for testing how neuronal activity influences axonal sprouting, circuit remodeling, and visual recovery, it does not by itself define the molecular programs or cell-type-specific states that enable regeneration. Therefore, single-cell and single-nucleus transcriptomic approaches are needed to identify the neuronal subtypes, glial states, and regeneration-associated gene programs that underlie injury-induced and activity-dependent axonal plasticity.

## 3 Single-cell RNA sequencing: decoding regenerative complexity

**FIGURE 3:**
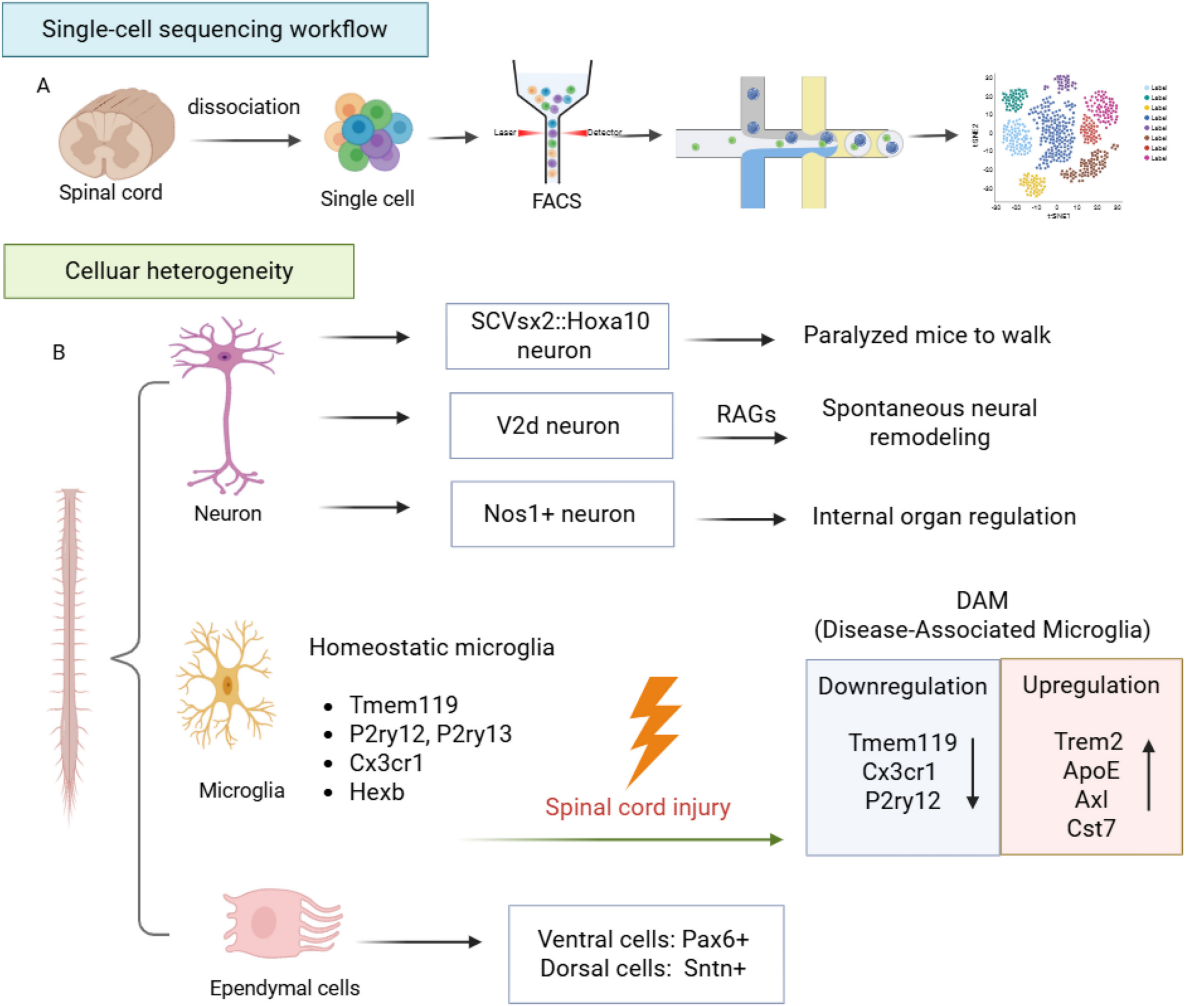
Single-cell RNA sequencing reveals cellular heterogeneity in the spinal cord. **(A)** Schematic workflow of scRNA-seq from tissue dissociation to sequencing. **(B)** Cellular heterogeneity analysis identifies key neural subtypes, including specific neuronal populations (e.g., SCVsx2::Hoxa10), disease-associated microglia (DAM) showing a characteristic gene expression shift after injury, and regionally distinct ependymal cell subtypes, providing insights into spontaneous neural remodeling and functional recovery.

Single-cell RNA sequencing (scRNA-seq) and single-nucleus RNA sequencing (snRNA-seq) have enabled high-resolution analysis of cellular heterogeneity, dynamic cell states, and injury-induced transcriptional programs in the CNS. Recent scRNA-seq and snRNA-seq studies have shifted the field from bulk descriptions of injured tissue to cell-type-specific mechanisms of regeneration. These studies have identified neuronal subtypes with distinct regenerative competence, injury-induced regeneration-associated gene programs, and microglia-dependent signaling networks that shape lesion homeostasis and axon regrowth after SCI and ONC (Brennan et al., 2022; Jacobi et al., 2022; Kathe et al., 2022; Li L. et al., 2022; Matson et al., 2022; Salvador et al., 2023). Representative applications of single-cell sequencing for identifying injury-responsive cellular heterogeneity in the spinal cord are summarized in Figure 3.

Despite these strengths, single-cell and single-nucleus sequencing have important technical and interpretive limitations that require careful consideration in axon regeneration studies. Tissue dissociation can introduce stress-response artifacts and may selectively lose fragile neuronal subtypes, reactive glial states, or long-projecting neurons that are particularly relevant to injury responses (Denisenko et al., 2020; van den Brink et al., 2017). Single-nucleus sequencing reduces some dissociation-related biases and is useful for frozen or difficult-to-dissociate CNS tissues, although it may capture transcriptomic features that differ from whole-cell scRNA-seq profiles (Cuevas-Diaz Duran et al., 2022). In addition, dropout effects and low transcript-capture efficiency can obscure low-abundance regeneration-associated genes, RNA-binding proteins, or locally translated axonal mRNAs (Jovic et al., 2022; Kharchenko et al., 2014). Conventional scRNA-seq also removes cells from their spatial context, making it difficult to determine whether injury-responsive cell states are located at the lesion core, lesion border, distal axon segments, or regenerating axon trajectories (Adema et al., 2024; Longo et al., 2021). Batch effects, differences in injury severity, time points, species, and sequencing platforms can further complicate comparisons across studies (Denisenko et al., 2020; Jovic et al., 2022). Therefore, single-cell data should not be interpreted as direct evidence of functional regeneration by itself. Integration with spatial transcriptomics, proteomics, genetic perturbation, optogenetic manipulation, and 3D imaging is needed to distinguish correlative injury signatures from causal regeneration mechanisms (Longo et al., 2021; Molla Desta and Birhanu, 2025; Renthal et al., 2020).

Beyond transcriptional profiling, recent studies also highlight the importance of post-transcriptional regulation in axon regeneration (Schaeffer and Belin, 2024). Local axonal RNA localization and translation allow injured axons to rapidly remodel their local proteome without relying entirely on somatic transcription, thereby supporting growth cone responses, cytoskeletal remodeling, metabolic adaptation, and axon regrowth (Le Coz et al., 2024; Taylor and Nikolaou, 2024). RNA-binding proteins further regulate the transport, stability, and translation of axonal mRNAs, linking RNA metabolism to regeneration-associated structural plasticity (Le Coz et al., 2024). Because transcriptomic changes do not always predict protein-level responses, translational control may provide an additional regulatory layer for CNS axon regeneration (Schaeffer and Belin, 2024). Recent work also showed that repeat-element RNAs can integrate neuronal growth programs through axonal mRNA polyadenylation and local translation, further supporting the relevance of RNA-based regulatory mechanisms in neuronal regeneration (Zahavi et al., 2025).

### 3.1 Cell-type-specific mechanisms of axonal regeneration in the spinal cord

Although single-cell studies have revealed broad cellular heterogeneity after SCI, their relevance to axonal regeneration lies primarily in identifying cell-type-specific mechanisms that regulate axon regrowth. This section therefore focuses on neuronal subtype-specific regenerative competence, regeneration-associated gene programs, axon remodeling, and neuron–glia interactions that directly influence axonal repair.

#### 3.1.1 Microglial heterogeneity in SCI

Microglia are resident immune cells of the CNS and respond rapidly to spinal cord injury. Earlier M1/M2 classifications have been replaced by single-cell-defined injury-responsive microglial states, which better capture the dynamic and heterogeneous microglial responses after SCI. In the context of axonal regeneration, microglial heterogeneity is most relevant through its effects on lesion homeostasis, extracellular matrix remodeling, and neuron–glia signaling. After SCI, microglia shift from homeostatic states marked by P2ry12, Cx3cr1, and Tmem119 toward injury-responsive states expressing Apoe, Spp1, Trem2, and related genes. Importantly, neonatal microglia can promote scar-free repair by depositing fibronectin-rich extracellular matrix bridges that support axon regrowth. In parallel, neonatal microglia-enriched proteinase/protease inhibitors may preserve a repair-permissive lesion environment by limiting excessive matrix degradation and inflammatory tissue damage (Brennan et al., 2022; Li et al., 2020). In adult SCI, microglia-dependent signaling networks coordinate interactions among CNS-resident glia and infiltrating immune cells, contributing to lesion homeostasis, tissue repair, and functional recovery (Brennan et al., 2022; Li et al., 2020; Salvador et al., 2023). Together, these findings indicate that microglia influence axonal regeneration not simply by inflammatory activation, but by shaping the structural and signaling environment of the injured lesion.

#### 3.1.2 Neuronal diversity in SCI

Single-cell and single-nucleus transcriptomic studies have identified spinal neuronal subtypes with distinct injury responses and regenerative competence. In the context of epidural electrical stimulation (EES)-mediated recovery, snRNA-seq and spatial transcriptomics identified a population of excitatory lumbar interneurons expressing Vsx2 and Hoxa10 (SC with Vsx2::Hoxa10) that is required for walking recovery after paralysis in corresponding EES with REHAB mouse models (Kathe et al., 2022). This finding links a defined neuronal subtype to circuit-level functional recovery after SCI.

Another study profiled cell types and dynamic responses in lumbar spinal cord neuronal subtypes across acute, subacute, and chronic stages after thoracic injury in mice. In this model, rare neuronal populations expressing regeneration-associated genes, including V2d neurons and spinocerebellar neurons, showed injury-induced transcriptional programs associated with spontaneous axonal remodeling, axon outgrowth, and collateral formation (Matson et al., 2022). Recent work in adult zebrafish further supports the importance of cell-type-specific repair programs by using snRNA-seq to identify intersecting modes of neuronal repair during innate spinal cord regeneration, including regenerative neurogenesis and neuronal plasticity programs that contribute to functional spinal cord repair (Saraswathy et al., 2024). This zebrafish study provides a regeneration-permissive vertebrate framework for comparing repair programs across species and injury contexts.

Together, these findings indicate that regenerative potential is not uniform across spinal neurons but is encoded by subtype-specific molecular programs. In addition, a single-nucleus transcriptomic atlas of the adult mouse spinal cord profiled 43,890 nuclei and identified 16 clusters of sympathetic motor neurons, including Nos1-expressing visceral motor neurons located in the lateral autonomic columns of the thoracic and sacral spinal cord (Sahel et al., 2021). Such cellular atlases provide a framework for linking neuronal diversity to motor function and injury-induced repair mechanisms.

#### 3.1.3 Developing human spinal cord

Although functional heterogeneity has been well characterized in the rodent spinal cord, whether similar cellular diversity exists in the human spinal cord remains an important question. The regulation of cell fate specification in the human spinal cord remains largely unknown, and understanding the spinal cord is essential to elucidate how disorders progress.

Spatial transcriptomics enables the visualization of RNA sequencing data and two-dimensional tissue section position information by employing barcode mapping to unique locations (Nature Methods, 2020). Integration of single-cell sequencing technology with spatial transcriptomics has been extensively used for investigating multi-omics transcriptomes at the single-cell level, particularly in elucidating the diverse origins associated with human fetal development and disease (Moncada et al., 2020). Integrated snRNA-seq and spatial multi-omics analysis of human prenatal spinal cord samples from post-conceptional weeks 5–12 revealed that active neural stem/progenitor cells enter a quiescent state earlier in human spinal cord development than in comparable rodent developmental stages (Li X. et al., 2023). This finding highlights species-specific timing in neural progenitor proliferation and cell fate specification during spinal cord development. Simultaneously, further investigation compared the diversification process of motor neurons and revealed several distinctions between mice and humans, demonstrating that α and γ motor neuron diversification occurs early in fetal development, while in mice it takes place at embryonic day 17.5 (Andersen et al., 2023).

The population of neural progenitor cells in the ventricular zone of the spinal cord undergoes significant changes during embryonic development and differentiates into ependymal cells with cilia during the fetal stage (Zhang et al., 2021). Mouse ependymal cells, which are stem cells for the spinal cord, possess a latent ability to differentiate into oligodendrocytes in order to remyelinate axons surrounding an injury (Llorens-Bobadilla et al., 2020). Ependymal cells in the spinal cord of mice are transcriptionally heterogeneous, and mature ependymal cells increase with age. Lateral ependymal cell maturation is reversible, but rapidly regains in injured spinal cord. In the adult human spinal cord, ependymal cells express the mature cell marker *Sntn* and uniquely co-express the lateral and ventral cell markers *Pax6* and *Arx* (Rodrigo Albors et al., 2023). Additionally, a comprehensive dynamic transcriptome of neural stem cells (NSCs) reveals that Nestin+ positive cells outside the central canal are NSCs and could potentially be used as endogenous NSCs for regenerative treatment of SCI (Shu et al., 2022).

### 3.2 Heterogeneity in the retina

The retina, located at the back of the eye, is composed of three cellular layers and consists of seven different cell types. It serves as the neural region where visual stimuli are detected and processed before being transmitted to the visual cortex. Due to its accessibility and observable nature, as well as its neuronal diversity, it provides an ideal model for studying cell development, neuronal differentiation, and the diversity of the nervous system (Masland, 2012). In past years, cells have been classified based on their molecular markers, morphological features, spatial organization, and biological functionality using conventional techniques such as immunofluorescence, electron microscopy, and electrophysiology. In the retina, the transcriptome profiles of many cell subtypes, especially rare cells, are often obscured by bulk RNA sequencing. Sorting and purification methods based on cell surface markers have not been developed to enrich all cell types. Consequently, retinal cells are primarily categorized into seven distinct types including bipolar cells (BCs), amacrine cells (ACs), retinal ganglion cells (RGCs), Müller glial cells, horizontal cells, and rod and cone photoreceptor cells (Hoon et al., 2014). The structure of the retina is extremely fine and complex, and the diversity of cell types as well as their functions are not fully understood. The emergence of scRNA-seq enables accurate classification and characterization of retinal cells in vitro and in vivo. Cellular RNA sequencing reveals the complexity of tissues by mapping single-cell transcriptomes, identifying cell-specific markers, discovering rare cell types and subtypes, and uncovering expression differences among retinal cells (Papalexi and Satija, 2018). Consequently, it is estimated that the retina harbors a diverse array of over 130 distinct neuronal cell subtypes (Dharmat et al., 2020; Lukowski et al., 2019).

The most common methods used in retina investigation are Drop-seq, 10x genomics, and Smart-seq2. For instance, Drop-seq was used for identifying whole subtypes populations in the retina and specific retinal bipolar neurons (Shekhar et al., 2016). The 10x Genomics was used to explore the cell type atlas of the human retina associated with age-related macular degeneration. Smart-seq2 enables *de novo* genome assembly and sequencing of transcripts in their entirety with fewer cells, providing detailed information on specific cell types or regions, particularly subtle differences between regenerating and non-regenerating RGCs. Consequently, a recent finding discovered regeneration-associated genes that significantly promote axon regeneration in mouse RGCs (Li L. et al., 2022).

Recent findings have greatly increased the number of analyses at the single-cell level applied to study retinal models, including mouse (Gautam et al., 2021; Hoang et al., 2020; Jacobi et al., 2022; Li L. et al., 2022; Luu et al., 2023; Rheaume et al., 2018; Shekhar et al., 2016; Tran et al., 2019), zebrafish (Hoang et al., 2020), chick (Hoang et al., 2020), macaque (Gautam et al., 2021; Yi et al., 2020), fetal human (Li R. et al., 2023), adult human (Gautam et al., 2021; Huang et al., 2023; Yi et al., 2020), and patients (Li Z. et al., 2022; Rheaume et al., 2018; Voigt et al., 2019). This has provided insights into multiple species of cell subtypes and related disease gene pathways in ophthalmology clinical treatment. The application of scRNA-seq technology to classify mouse retinal cells is currently the most extensive and detailed. A study performed scRNA-seq on 44,808 retinal cells in mice, classifying them into ten main types with proportions such as BCs (14%) and RGCs (1%). Using t-SNE dimensionality reduction, these ten cell types were further divided into 38 subclasses, providing the most accurate systematic classification and definition of retinal cells at that time. This analysis offered characteristic expression profiles and precise proportions for each type of retina for the first time (Macosko et al., 2015). Aimed at mouse retinal bipolar cells, analysis of 25,000 single-cell transcriptomes revealed 15 subtypes, including all previously observed types and two new types, thus increasing the diversity beyond the previous eight subcategories. Interestingly, one of the two new types exhibits a unique morphology and position (Macosko et al., 2015; Masland, 2012; Shekhar et al., 2016). Amacrine cells (ACs) form a diverse group of interneurons positioned to modulate input from photoreceptors and transmit it to RGCs, making an important contribution to retinal signal processing, integration, and transmission (Masland, 2012). The ACs were categorized into 21 subclasses based on markers such as Maf. Furthermore, employing in situ hybridization and immunofluorescence techniques, a total of 63 subclasses were identified, encompassing several novel subtypes that were subsequently validated (Yan et al., 2020). Importantly, the expression of Meis2 (a marker of GABAergic cells) and Tcf4 (a marker of glycinergic cells) confirmed ACs as inhibitory neurons, highlighting their transcriptional relationships within closely related types.

### 3.3 Subtype-specific susceptibility: RGCs

Within the retina, photoreceptors transmit discernible visual signals to interneurons, which subsequently process and relay them to retinal ganglion cells (RGCs), constituting less than 1% of the total retinal cell population (Masland, 2012; Smith and Chauhan, 2015). Previous studies have classified RGCs based on their morphologies, physiologies, and molecules, with a particular focus on distinguishing around 30 types of RGCs (Sanes and Masland, 2015). Around the same time, researchers discovered that at least 32 different functional types of retinal RGCs in mice could be distinguished based on their electrophysiological properties. This suggests that molecular differences among these subtypes of RGCs may be associated with their distinct electrophysiological functions (Baden et al., 2016). For the first time, single-cell RNA-seq analysis of postnatal day 5 mouse retinas identified 40 subtypes of RGCs and their markers/transcription factors, including Zic1 for the right eye subtype and Runx1/Fst for a novel subtype confirmed by FISH fitting methods. Subsequently, a comprehensive molecular atlas of 46 types of retinal ganglion cells (RGCs) in the adult retina was analyzed using the scRNA-seq method (Tran et al., 2019). Furthermore, novel subtypes of RGCs, such as Col25a1+RGCs, have been identified, leading to a more precise definition and analysis of RGCs as crucial visual signaling cells.

In adult animal CNS, axonal regeneration and synapse formation are inherently limited, leading to persistent functional deficits following axonal injury. To address this formidable challenge, researchers have employed axonal injury models to identify molecules that augment axonal regeneration and neuronal survival. Currently, optic nerve crush (ONC) serves as a widely utilized model for investigating the mechanisms underlying CNS regeneration. Following ONC in mice, approximately 70% of retinal ganglion cells (RGCs) undergo cellular phagocytosis-mediated cell death within 8 days, while nearly 90% of RGCs succumb within 1 month post-injury (Tran et al., 2019). Only a small fraction of regenerating axons extend beyond the lesion site by several hundred micrometers (Winter et al., 2022).Notably, all subtypes of RGCs maintain expression of Rbpms genes up to 14 days post crush.

Regenerative vertebrate models such as zebrafish provide an important contrast to mammalian ONC models. Unlike adult mammalian RGCs, zebrafish RGCs are highly resilient to optic nerve injury and can mount robust regenerative responses. Recent single-cell analysis showed that zebrafish optic nerve injury is associated with broad RGC dedifferentiation and activation of progenitor-like and regeneration-associated gene programs across RGC subtypes (Ali et al., 2025). In adult zebrafish, Srebf2-mediated activation of the mevalonate synthesis pathway was further shown to promote optic nerve axon regeneration and visual functional recovery (Hu and Veldman, 2025). In larval zebrafish, live imaging has also revealed glial-derived guidance mechanisms that direct regenerating RGC axons toward the optic chiasm, highlighting the value of transparent regenerative models for studying axon pathfinding and target reconnection (Harvey et al., 2026)

**FIGURE 4:**
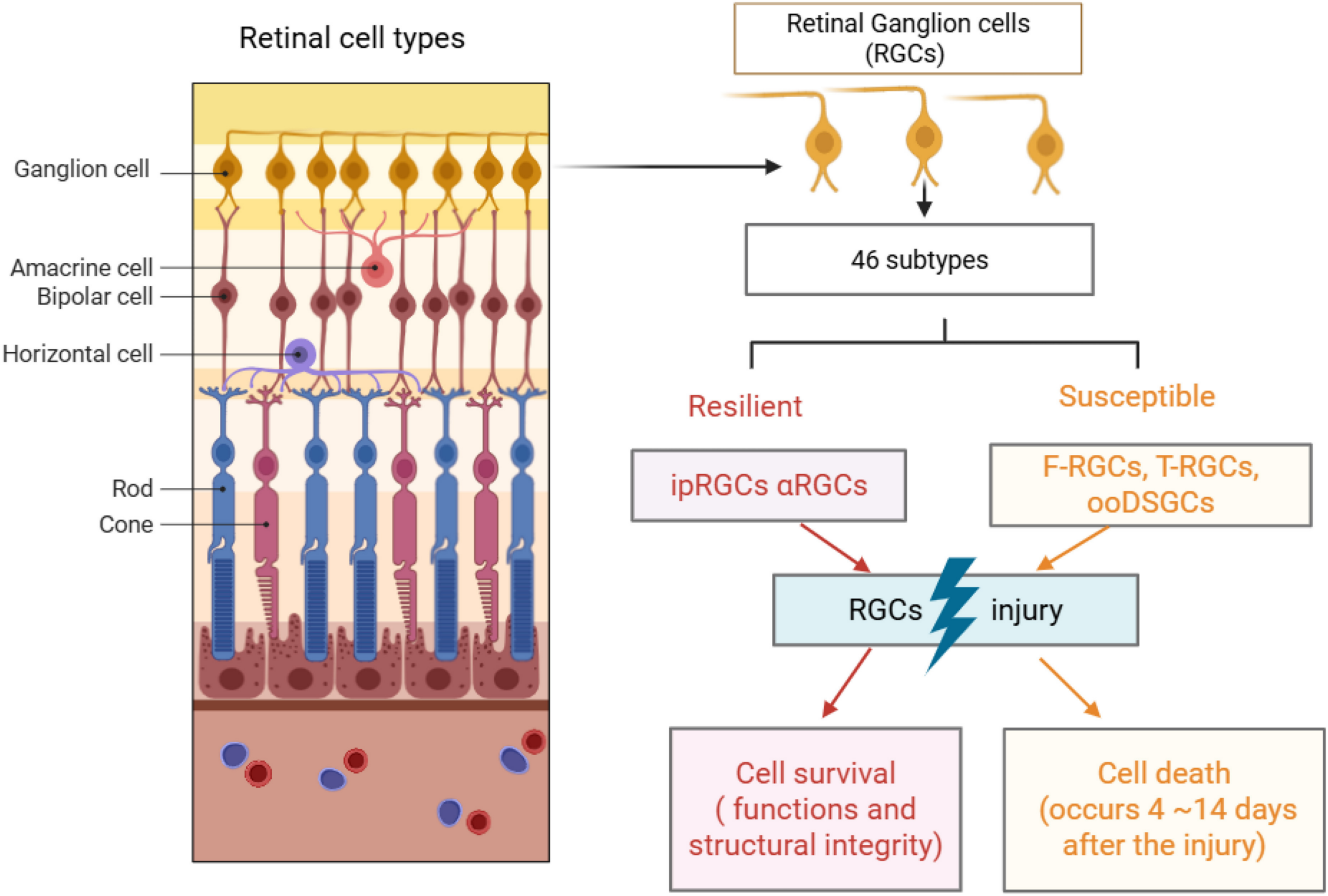
Heterogeneity of RGC subtypes in response to injury. scRNA-seq reveals 46 molecularly distinct retinal ganglion cell (RGC) subtypes exhibiting differential vulnerability to optic nerve injury, with resilient subtypes (e.g., ipRGCs and αRGCs) surviving while susceptible subtypes (e.g., F-RGCs and ooDSGCs) undergo rapid degeneration, mediated by subtype-specific genes including Opn4 and Spp1.

Studies have provided a basis for categorizing distinct gene-marker-expressing RGC subtypes in mice. These subtypes exhibit marked differences in susceptibility to optic nerve injury (Figure 4; Jacobi et al., 2022; Li L. et al., 2022; Tran et al., 2019). Indeed, survival rates vary widely among different subtypes of RGCs. Alpha-RGCs (αRGCs) are one of the largest subtypes of RGCs, characterized as a resilient subclass that can survive for months after ONC. Notably, αRGCs are involved in nearly all processes associated with the phosphatase and tensin homolog (PTEN) downregulation to activate the mTOR pathway (Duan et al., 2015). It indicates that endogenous levels of mTOR in RGCs subtypes may contribute to the susceptibility of certain RGC subtypes to injury. The αRGCs subclass selectively expresses osteopontin (OPN) and receptors for insulin-like growth factor 1 (IGF-1). Combining OPN with IGF-1 can effectively promote regeneration, comparable to PTEN down-regulation (Duan et al., 2015). However, notable differences in resilience exist among the various subtypes of αRGCs. Despite their close transcriptional proximity, the two transient types aRGCs exhibit higher susceptibility compared to the two sustained highly resilient types (Duan et al., 2015).

It indicates that RGCs subtypes differ in their sensitivity to injury and endogenous levels of mTOR in RGCs subtypes may lead susceptibility of RGC subtypes to injury. Intrinsically photosensitive RGCs (ipRGCs) types, defined by the expression of *Opn4*, were resilient to ONC (Krieger et al., 2017; Liu et al., 2018). Moreover, *Timp2* was selectively enriched in resilient ipRGCs, while *Mmp12* was broadly upregulated in modest ipRGCs after ONC.

Axon regeneration is a complex process that involves the coordination of multiple RAGs in response to transcriptional changes, such as PTEN, SOCS3, and CNTF (Park et al., 2008; Pernet et al., 2013a; Smith et al., 2009). According to recently described criteria, gene expressions in each of the 46 types of RGCs were assessed using single-cell transcriptomics after PTEN knockout, SOCS3 knockout, CNTF overexpression, or a combination treatment following ONC injury (Jacobi et al., 2022; Tran et al., 2019). It has been found that the combined manipulation improves cell survival across most types of RGCs, and the degree of neuroprotection is generally associated with the inherent resilience of different cell types. Interestingly, there were slight changes in gene expression during the initial 2 days and stable expression between 7 and 21 days after ONC. In contrast, gene expression for survival and regeneration significantly increases between 2 and 7 days following ONC, indicating a window of time for nerve regeneration treatment (Jacobi et al., 2022). Single-cell analysis of regenerating and surviving RGCs after ONC identified dynamic transcriptional programs associated with axon regrowth and neuronal survival. This work highlighted candidate regeneration-associated genes, including *Anxa2*, *Plin2*, *Mpp1*, *Acaa2*, *Spp1*, and *Lgals1*, which are involved in lipid metabolism, cell adhesion, migration, and axon regeneration (Li L. et al., 2022). These genes were found to promote optic nerve regeneration to varying extents.

More recent studies have extended these findings by identifying intracellular mechanisms that couple RGC survival with optic nerve regeneration. Yang Hu and colleagues showed that OPTN facilitates microtubule-dependent axonal mitochondrial transport through the TRAK1/KIF5B machinery, and that restoring OPTN/TRAK1/KIF5B signaling promotes RGC neuroprotection and optic nerve regeneration (Liu et al., 2025). In addition, actin depolymerization was shown to promote axon regeneration by restoring axonal mitochondrial transport in mouse models of optic neuropathy (Li et al., 2026). GPX4-mediated suppression of lipid peroxidation was also reported to promote optic nerve regeneration and RGC neuroprotection (Yang et al., 2026). These findings suggest that successful optic nerve repair requires not only activation of classical growth pathways or regeneration-associated transcriptional programs, but also restoration of axonal mitochondrial delivery, cytoskeletal remodeling, and resistance to lipid-peroxidation-associated stress.

Together, these studies show that single-cell transcriptomics can identify regeneration-associated genes, whereas recent mechanistic studies further reveal downstream cellular processes that regulate RGC survival, axonal transport, and optic nerve regeneration.

### 3.4 Human retina and its organoids

The human retina is a complex tissue made up of different light-sensitive cells, and understanding its development, differentiation, and genetic changes in disease states is crucial for studying retinal pathologies. For the first time, researchers profiled human retinal cells using the scRNA-seq platform and identified human retinal astrocytes through three subtype-specific markers: FOS, FTL, and COL4A3, along with their corresponding gene expression patterns (Menon et al., 2019). Nearly at the same time, a study profiled the human retina through unsupervised clustering analysis of transcriptome atlases from three donors and identified 18 transcriptionally distinct cell populations representing all neural retinal cells. The retina transcriptome atlas provided unprecedented insights into the transcriptional landscape of human retinal cells, particularly focusing on benchmark pluripotent stem cell-derived cone photoreceptors and an adult Müller glial cell line (Lukowski et al., 2019). More recently, a combination of transposase-accessible chromatin sequencing (scATAC-seq) and scRNA-seq assays performed on human embryonic eye samples between 9 and 26 weeks gestation revealed that MGC2 interacts highly with cones and plays a crucial role in the normal development of neurons in the macular area of the retina during embryonic development (Li R. et al., 2023). Not only in human retinal tissues, but also in aging non-human primate cynomolgus monkey retina that scRNA-seq analysis was conducted, defining 15 subclasses of cells with specific gene expression characteristics. Additionally, a Muller cell subclass with neural developmental potential was identified. Furthermore, analysis of differentially expressed genes (DEGs) showed that the inflammatory response in retinal pigment epithelium and choroid cells was significantly enhanced, suggesting a relationship between oxidative stress and inflammation in retinal aging (Wang et al., 2021).

For further investigation of retinal pathology, scientists have also identified 34 risk loci associated with age-related macular degeneration (AMD), indicating that cone photoreceptors, glial cells, and vascular cell types are likely to be more susceptible to genetic perturbations affecting AMD susceptibility (Menon et al., 2019). Similarly, single-cell transcriptomics were conducted on macular and peripheral regions of the retinal pigment epithelium choroid from 7 human donor eyes, which are closely associated with the pathogenesis of AMD. This study revealed that in response to complement activation, the choriocapillaris exhibits a high and specific expression of a cell cycle regulatory gene (Voigt et al., 2019).

Currently, the lack of animal models for retinal diseases poses a challenge in retinal research. Human organoids recapitulating the cell-type diversity and function of their target organ are valuable for basic and translational research, opening the window for clinical diagnose and treatments. More Intriguingly, human embryonic stem cells can induce retinal organoids, and the intervention of retinal organoids to construct related retinal disease models benefits research on retinal function and diseases. Subsequently, in the context of human retinal development, single-cell RNA sequencing has been applied to study how the cell types of human fetal retinal differentiation compare with those in corresponding stages of retinal organoids (Finkbeiner et al., 2022; Hu et al., 2020; Lu et al., 2020; Sridhar et al., 2020).

Human pluripotent stem cell (hPSC)-derived retinal organoids are regarded as potential models for retinal degeneration. A scRNA-seq study comparing hPSC-derived retinal organoids with stage-matched fetal human retina revealed both substantial similarities and important differences in gene expression and cell-state composition (Finkbeiner et al., 2022). For example, ATOH7 is more highly expressed in fetal retinal cells than in organoids, whereas GADD45A is more highly expressed in organoids (Sridhar et al., 2020). Furthermore, the analysis of scATAC-seq revealed a high correlation in chromatin accessibility between organoids and human fetal retinas, as well as transcription factors relevant to specific cell fate. Notably, the role of Notch signaling differs in cell composition between organoids and fetal retina (Finkbeiner et al., 2022). In fact, retinal organoids *in vitro* and retinas *in vivo* were found to develop at similar rates (Cowan et al., 2020). Importantly, temporal and spatial rules governing the developmental sequence and differentiation of the main human retinal cell types were delineated through scRNA-seq analysis for the first time. The transcriptional networks were conducted on human embryonic to adult retinas at 16 different time points, as well as on human retinal organoids at 4 developmental stages, defining approximately 120,000 captured cells classified into 126 subclasses (Lu et al., 2020).

Above all, ATOH7 has been believed to be the key factor regulating the differentiation and development of retinal cells (Finkbeiner et al., 2022; Lu et al., 2020; Sridhar et al., 2020). *Atoh7* (atonal bHLH transcription factor 7, known as Math5) is transiently expressed in the mouse retina at E11 and serves as the primary neurogenic transcription factor required for RGC development (Brown et al., 2001; Wang et al., 2001). Furthermore, using the chicken retina as a model, it has been shown that cell autonomous expression of *Atoh7* is sufficient to induce precocious RGC formation and expansion of the neurogenic territory (Zhang X. M. et al., 2018). Recently, single-cell RNA sequencing of *Atoh7;Bax*-deficient retinas reveals delayed RGC differentiation, but normal gene expression profile of RGC precursors. However, these cells show severe axonal guidance defects in retinal circuitry integration, highlighting the essential role of *Atoh7* in facilitating proper axon guidance of the nascent axons of differentiating RGCs (Brodie-Kommit et al., 2021). Thus, ATOH7 provides insight into manipulating the production of RGCs from stem cell-derived retinal organoids by promoting early retinal genesis and specifying the RGC differentiation program. The major findings from single-cell transcriptomic analyses of the spinal cord and retina, including key cell types, technical methods, and regeneration-associated discoveries, are summarized in Table 1.

**TABLE 1:** Applications of single-cell sequencing in CNS regeneration.

Tissue	Cell types	Technical methods	Core findings and conclusions	References
Spinal cord	Microglia	scRNA-seq, snRNA-seq	Discovered disease-associated microglia (DAM) phenotype regulated by the Trem2-ApoE pathway. After SCI, microglia downregulate homeostatic genes (e.g., P2ry12, Tmem119) and upregulate DAM genes (e.g., ApoE, Spp1). Aging impairs microglial state transition capacity.	(Keren-Shaul et al., 2017; Krasemann et al., 2017; Deczkowska et al., 2018; Li et al., 2020; Brennan et al., 2022; Gao et al., 2023; Salvador et al., 2023)
Spinal cord	Neurons	snRNA-seq Spatial Transcriptomics	Identified key excitatory interneuron subpopulations (e.g., VSX2::Hoxa10) mediating recovery of walking after paralysis. Discovered neuronal subtypes expressing rare regeneration-associated genes (RAGs, e.g., V2d neurons) that promote spontaneous axonal remodeling.	(Kathe et al., 2022; Matson et al., 2022)
Spinal cord	Ependymal cells	scRNA-seq, snRNA-seq	Revealed transcriptional heterogeneity and potential stem cell properties of ependymal cells in adult mouse and human spinal cords. Injury can reverse their maturation state, suggesting regenerative potential	(Zhang et al., 2021; Shu et al., 2022; Rodrigo Albors et al., 2023)
Spinal cord	Neural progenitors	snRNA-seq Spatial Multi-Omics	Constructed spatiotemporal gene expression maps of human embryonic spinal cord development. Revealing species-specific differences in α/γ motor neuron differentiation.	(Andersen et al., 2023; Li X. et al., 2023)
Retina	Retinal ganglion cells	snRNA-seq (10x Genomics, Smart-seq2)	Systematically identified 46 RGC subtypes. Revealed differential vulnerability among subtypes (e.g., αRGCs and ipRGCs are more resilient). Discovered regeneration-associated genes (e.g., Anxa2, Spp1) that promote axon regeneration. Combinatorial intervention (PTEN/SOCS3 knockout + CNTF) enhances survival across most RGC subtypes.	(Duan et al., 2015; Rheaume et al., 2018; Tran et al., 2019; Jacobi et al., 2022; Li L. et al., 2022)
Retina	Bipolar cells	Drop-seq	Comprehensive classification of mouse retinal bipolar cells revealed 15 subtypes, exceeding previous morphological categories.	(Macosko et al., 2015; Shekhar et al., 2016)
Retina	Amacrine cells	scRNA-seq	Identified 63 amacrine cell subtypes, highlighting immense diversity. confirmed inhibitory neuron identity using markers (e.g., Meis2, Tcf4).	(Yan et al., 2020)
Retina	Retinal pigment epithelium	scRNA-seq, scATAC-seq	Established single-cell atlases of human fetal and adult retinas, defining all major cell types. Revealed subtle but important differences between retinal organoids and native retinas in developmental pace, cell composition (e.g., Notch signaling), and gene expression (e.g., ATOH7).	(Lukowski et al., 2019; Menon et al., 2019; Cowan et al., 2020; Sridhar et al., 2020; Finkbeiner et al., 2022; Li R. et al., 2023)
	Human retinal organoids	scRNA-seq	Found that cone photoreceptors, glial cells, and vascular cells are likely the primary carriers of genetic risk for age-related macular degeneration (AMD). Revealed specific responses of the choriocapillaris to complement activation.	(Menon et al., 2019; Voigt et al., 2019)

Although single-cell and spatial transcriptomic approaches have substantially improved the resolution of cell identity and spatial organization in regenerative contexts, they cannot by themselves determine whether newly growing axons follow appropriate three-dimensional trajectories or reinnervate relevant targets. This gap is particularly important in CNS injury, where increased axon growth does not necessarily correspond to accurate pathfinding or functional circuit repair. Tissue clearing and 3D imaging therefore provide a complementary structural framework for assessing axonal morphology, long-range trajectories, branching patterns, growth cone behavior, and potential circuit reconstruction in intact tissues.

## 4 Tissue clearing: visualizing neuronal networks in 3D imaging

**FIGURE 5:**
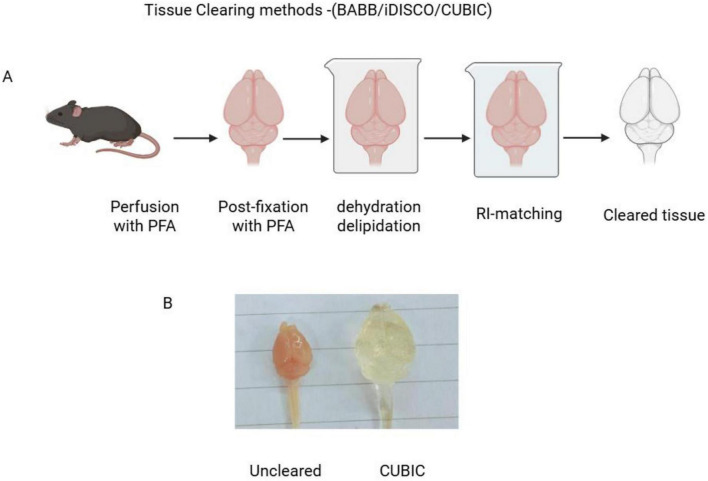
Workflow and effect of tissue-clearing methods for 3D imaging. **(A)** Schematic procedure of solvent-based tissue clearing (e.g., BABB, iDISCO, or CUBIC), including steps such as fixation, dehydration, and refractive index matching, to render tissues transparent. **(B)** Comparison of uncleared and CUBIC-cleared tissue, demonstrating enhanced depth-independent imaging capability for whole-organ structural and molecular analysis.

Biological tissues are inherently opaque, and conventional two-dimensional histological sectioning provides only fragmented spatial information about axonal trajectories and neural circuit architecture. Tissue clearing, when combined with light-sheet or confocal 3D imaging, enables visualization of intact neural tissues without serial sectioning and allows axonal projections and injury-induced structural changes to be analyzed in their native spatial context (Figure 5). Different clearing methods, including CLARITY, CUBIC, iDISCO, 3DISCO, and HYBRiD, vary in clearing chemistry, processing time, fluorescence compatibility, and suitability for different tissue types. Their technical features, best-suited applications, key limitations, and relevance to axon regeneration studies are summarized in Table 2.

**TABLE 2:** Comparison of major tissue clearing methods for 3D imaging.

Method	CLARITY	CUBIC	BABB
Core mechanism	Hydrogel-embedding: Proteins are cross-linked to a polyacrylamide hydrogel scaffold. Lipids are then removed via electrophoresis or passive incubation	Chemical reagent treatment: uses aminoalcohols for delipidation and decolorization, and high-refractive index agents (e.g., Histodenz, sucrose) for refractive index matching.	Organic solvent dehydration: Tissue is dehydrated through an ethanol gradient and then cleared with the organic solvent BABB (a 1:2 mixture).
Clearing medium	Aqueous solutions (e.g., FocusClear, RIMS)	Aqueous solutions (e.g., CUBIC-mount)	Organic Solven (BABB mixture)
Processing time	Long (days to week)	Medium (Several days to 1–2 weeks)	Fast (hours to 2 days)
Tissue morphology/size change	Excellent morphology preservation, minimal shrinkage	Tissue expands significantly (up to 2x original volume)	Tissue shrinks considerably (∼30–50%)
Sample compatibility	Large samples (e.g., whole brain, spinal cord)	Large samples (e.g., whole brain, whole body embryos); excellent decolorization	Small-to-medium samples (< 1 mm thick)
Fluorescent protein compatibility	Excellent (Aqueous process preserves most fluorescent proteins well)	Good (Aqueous, but high detergent concentrations may quench some FPs)	Poor (Organic solvents destroy most fluorescent proteins; best for immunolabeling or chemical dyes)
Key advantages	1. Best structural integrity 2. Superior FP compatibility 3. Compatible with immunostaining 4. Ideal for connectomics	1. Powerful decolorization 2. Simpler protocol (passive) 3. Excellent clearing depth 4. Enables whole-body clearing	1. Rapid protocol 2. Low cost 3. Excellent clarity for small samples 4. Well-established, classic method
Best suited for	Thick CNS tissues requiring preservation of endogenous fluorescence, tissue morphology, and immunolabeling.	Large tissues or whole organs requiring deep imaging, strong decolorization, and scalable clearing.	Small-to-medium samples requiring rapid clearing and high optical transparency.
Key limitations	Long processing time; requires protocol optimization; clearing may be incomplete in dense adult CNS tissues.	Tissue expansion may affect precise morphometric measurements; high detergent concentrations may reduce fluorescent protein signals.	Considerable tissue shrinkage; poor endogenous fluorescent protein preservation; less suitable for samples requiring native fluorescence.
Relevance to axon regeneration studies	Useful for preserving axonal morphology, lesion architecture, glial scar organization, and immunolabeled regeneration-associated markers.	Useful for large-scale 3D visualization of spinal cord or brain-wide axonal projections, lesion environments, and tissue-wide repair responses.	Useful for rapid visualization of labeled axonal tracts, long-range trajectories, and gross regeneration patterns in smaller samples.

Beyond anatomical visualization, tissue clearing and 3D imaging provide structural insight into axonal regeneration by revealing the spatial trajectories of regenerating axons in intact tissues. In spinal cord and optic nerve injury models, 3D imaging can uncover axonal misguidance, U-turns, stalled growth cones, and aberrant branching, features that are easily missed in conventional 2D sections. These observations emphasize that increased axon growth does not necessarily indicate accurate pathfinding, target reinnervation, or functional circuit repair.

These advantages are particularly relevant for distinguishing true axon regeneration from spared or incompletely injured axons, a major challenge in conventional histological analyses. Although tissue clearing enables high-resolution endpoint visualization of axonal trajectories, longitudinal *in vivo* imaging provides a complementary strategy to follow the same axons over time after injury. In transparent vertebrate models such as larval zebrafish, live imaging can directly track axonal regrowth, guidance, and functional recovery after SCI (Becker et al., 2023; Hecker et al., 2020). Adult Danionella cerebrum further extends this advantage to an optically accessible adult vertebrate model, allowing repeated *in vivo* imaging over days to weeks (Lam, 2022). These approaches help distinguish true axon regeneration from spared or uninjured axons.

Indeed, the emerging tissue clearing methods enable unbiased visualization of the 3D anatomy of axonal tracts in the spinal cord without the need for tissue sectioning (Wu et al., 2022). A tetrahydrofuran (THF)-based clearing procedure was first used to render fixed and intact spinal cord tissue transparent for 3D imaging after SCI. It was found, over a year post-injury, that growth-competent axons showed substantial regrowth through the injury site, as evidenced by fluorescently labeled cells and two-photon microscopy. Additionally, a few growth-incompetent axons were able to regenerate by bypassing the lesion (Hilton et al., 2019; Xie et al., 2023). Additionally, the study utilized 3DISCO clearing to examine axon regeneration following incomplete SCI. By injecting AAV, they successfully traced two distinct supraspinal pathways, the CST and RST, and visualized their bundles as well as collateral branch trajectories in cleared spinal cord segments (Soderblom et al., 2015). Recently, comprehensive tissue clearing, tissue expansion, and tiling light sheet microscopy methods have been developed to image the large genetically labeled mouse spinal cord locomotor neural network from the cellular to synaptic level with high throughput. Importantly, the utilization of high-throughput 3D imaging to visualize intact axonal tracts holds significant potential in assessing the efficacy of experimental therapies for spinal cord injury and other neurological disorders.

Recent investigations have identified several strategies, including deletion of growth repressors such as PTEN (Jacobi et al., 2022; Park et al., 2008), SOCS3 (Jin et al., 2015; Smith et al., 2009), KLF4 (Moore et al., 2009), and UTX (Yang et al., 2025), that can induce long-distance regeneration in the optic nerve after injury. A key question is whether regenerating RGC axons can successfully navigate toward their appropriate targets. In the past, optic nerve imaging in rodent models required generating hundreds of consecutive tissue sections, which resulted in fragmented information about axonal trajectory. Consequently, morphometric measurements of axonal regeneration location and termination could lose precision and accuracy. In general, tissue clearing enables the 3D imaging of the entire optic nerve, allowing visualization of the true morphology of regenerating axons (Luo et al., 2013; Wang et al., 2018; Wang et al., 2020). A THF-based clearing method combined with light sheet fluorescence microscopy (LSFM) allows for deep tissue fluorescence imaging in unsectioned optic nerves. It has been observed that axonal regeneration in PTEN/SOCS3 KO mice follows a convoluted path, making multiple turns and extending back to the eye (Luo et al., 2013). Similarly, 3D imaging analysis revealed that stimulation of regenerating RGC axons with AAV2 containing constitutively active Stat3 (Stat3-ca) resulted in elongation beyond the lesion site and frequent U-turns, indicating significant issues with directionality and guidance in the injured optic nerve (Pernet et al., 2013b). Furthermore, tissue clearing and confocal imaging of whole-mount optic nerves enable us to visualize the native growth cone morphology and axon trajectory after optic injury. Additionally, analysis of 3D imaging reveals that deletion of myosin IIA/B in mice abolishes the formation of retraction bulbs and efficiently enhances RGC axon regeneration. Together, these results highlight the significance of tissue clearing and 3D imaging in detecting axon morphology and trajectory, which would have been challenging to identify in histological sections.

Together, these examples demonstrate that tissue clearing and 3D imaging can reveal previously unrecognized aspects of axonal regeneration that are difficult to infer from conventional 2D histology. These include convoluted axonal trajectories, U-turns, growth cone morphology, retraction bulb formation, aberrant branching, and pathfinding errors in injured optic nerves. Therefore, 3D imaging can determine not only whether axons regrow, but also whether regenerating axons follow appropriate trajectories for structural circuit repair. The major biological questions, strengths, limitations, and applications of optogenetics, single-cell/single-nucleus omics, and tissue clearing/3D imaging in CNS axon regeneration studies are summarized in Table 3.

**TABLE 3:** Comparative summary of key technologies for studying CNS axon regeneration and structural plasticity.

Technology	Main biological question	Major strengths	Key limitations	Key applications in CNS regeneration
Optogenetics	Does neuronal activity causally regulate axon growth, sprouting, circuit rewiring, and functional recovery?	High temporal precision; cell-type-specific activation or inhibition; enables causal functional testing	Limited light penetration; viral delivery barriers; possible phototoxicity or opsin overexpression; long-term safety concerns	Testing activity-dependent axon sprouting, growth cone dynamics, spinal circuit repair, retinal light responsiveness
Single-cell/single-nucleus omics	Which neuronal subtypes, glial states, and molecular programs are associated with regenerative competence?	Resolves cellular heterogeneity; identifies RAGs and injury-responsive states; useful for subtype-specific analysis	Dissociation artifacts; dropout effects; loss of spatial information; mostly correlative without functional validation	Identifying regeneration-associated genes, resilient RGC subtypes, microglial injury states, neuronal repair programs after SCI and ONC
Tissue clearing/3D imaging	Do regenerating axons follow correct trajectories and reconnect with appropriate targets?	Visualizes intact 3D tissue architecture; visualizes long-range axonal trajectories; detects U-turns, branching, growth cones, and pathfinding errors	Endpoint analysis; protocol complexity; tissue shrinkage or expansion; limited molecular/functional information alone	Mapping axonal trajectories, lesion architecture, growth cone morphology, optic nerve regeneration, spinal cord circuit reconstruction
Integrated framework	How can molecular mechanisms, functional activity, and structural repair be linked?	Connects molecular profiling, causal testing, and spatial validation	Requires multimodal experimental design and cross-platform integration	Single-cell omics identifies candidates; optogenetics tests function; tissue clearing validates structural repair

## 5 Conclusions and perspectives

Optogenetics enables precise temporal and spatial manipulation of biological processes and behaviors. However, to address potential limitations such as phototoxicity and protein overexpression associated with optogenetics, it is often necessary to validate its implementation in the corresponding biological context. Furthermore, while transgenic and gene-targeting techniques have been extensively developed for implementation in rodents, their application in primate models during clinical transition is still in a nascent stage. In the clinical therapies, optogenetics has primarily been limited to the treatment of retinal disorders, with ongoing research focusing on the management of hearing impairments and arrhythmias. Currently, the primary challenges in optogenetics encompass safety concerns, efficient gene and light delivery systems. In the future, the advancement of optogenetic technology will not only promote its broader application in central nervous systems but also facilitate investigations into disorders of the peripheral nervous system as well as non-neural tissue diseases in intricate animal models and humans.

Single-cell sequencing technology unveils cellular heterogeneity and functional disparities in the retina, spinal cord, their organoids, and other central nervous system tissues. Despite conspecific cells sharing similar genomes, they undergo changes in differential regulation of gene expression and metabolic pathways, which lead to differentiation in cellular functions during processes of development and regeneration. The integration of single-cell analysis with other multi-omics technologies, such as spatial transcriptome sequencing, is of great significance in elucidating the intricate processes of cellular diversity and unraveling the underlying mechanisms of neuronal degeneration and regeneration, eventually leading to a better understanding of diseases and clinical interventions. With continued advances in single-cell isolation and sequencing technologies, a large range of single-cell sequencing technologies have emerged, encompassing scChIP-seq, scATAC-seq, and other techniques (Baek and Lee, 2020; Ku et al., 2019). However, the effective integration of single-cell data from disparate studies poses significant challenges. Next, it is necessary to use statistical and computational methods to analyze the rules from a large number of single-cell sequencing data and conduct scientific verification through biological experiments.

Tissue clearing and 3D imaging represent an important advance over conventional 2D histology by enabling less biased visualization of histological information. Significantly, it facilitates the visualization of precise spatial trajectories and ultimate destinations of regenerating axons. However, the need for protocol optimization, specialized imaging infrastructure, and technical expertise hinders the widespread application of tissue transparency techniques. Additionally, the relatively low resolution of 3D imaging hampers the analysis of subcellular or cellular processes. Innovations in tissue transparency and large-scale light sheet microscopy are required to facilitate new applications for multi-dimensional imaging.

Notably, CRISPR genome editing has emerged as a powerful research tool in the field of biological science for precise genome editing in bacteria, model organisms, and human cells (Li T. et al., 2023; Pickar-Oliver and Gersbach, 2019). Recently, an array of CRISPR-Cas tools with an expanded repertoire of targets has been engineered and developed, including CRISPRa/CRISPRi for targeted transcriptional activation or interference (Fontana et al., 2020; Liu et al., 2019; Tong et al., 2020) and CRISPR-Cas13 for RNA editing (Abudayyeh et al., 2017; Cox et al., 2017). With the continuous iterative optimization of CRISPR gene editing technology, it has also demonstrated significant potential in the realm of gene therapy for diverse neurological disorders. For instance, the integration of CRISPR-based high-throughput *in vivo* functional screening with multi-omics analysis could help discover new genes that promote neural survival and regeneration (Tian et al., 2022). Therefore, CRISPR holds great promise as a high-throughput method for screening genes implicated in neuroprotection and regeneration. However, the application of gene editing technology, particularly in the context of clinical gene therapy, necessitates meticulous evaluation of ethical considerations and potential risks.

A useful way to interpret these technologies is to match each approach to the biological question it is best suited to answer. Optogenetics enables causal testing of neuronal activity and circuit function, but provides limited molecular and structural information by itself. Single-cell and single-nucleus sequencing can identify injury-responsive cell types, neuronal subtypes, and regeneration-associated molecular programs, but these approaches are largely correlative without functional validation. Tissue clearing and 3D imaging provide spatially intact visualization of axonal trajectories, growth cones, branching, and pathfinding errors, but they usually capture endpoint structural information rather than direct causal mechanisms. Thus, these approaches should be viewed as complementary rather than interchangeable tools. Key unresolved questions include which molecular programs are truly causal for regeneration, which neuronal subtypes have the highest intrinsic regenerative competence, how neuronal activity shapes axon growth and guidance, and whether structurally regenerated axons form target-specific functional reconnections.

Future studies will benefit from combining these technologies in the same experimental framework. For example, single-cell or single-nucleus sequencing, especially when integrated with spatial multi-omics, can first identify injury-responsive neuronal subtypes and candidate regeneration-associated pathways (Dong et al., 2025). Optogenetic manipulation can then test whether activating defined neuronal populations or activity patterns promotes axonal sprouting and circuit-level recovery, as recently shown for transcranial optogenetic stimulation of corticospinal tract regeneration through the JAK2/STAT3 pathway after SCI (Ma et al., 2025). Finally, tissue clearing and 3D imaging can determine whether increased axon growth corresponds to accurate long-range trajectories, target reinnervation, and structural circuit repair. This integrated strategy would move the field beyond descriptive profiling toward causal and spatially resolved mechanisms of CNS axon regeneration.

Collectively, optogenetics, single-cell sequencing, and tissue clearing technologies have substantially advanced the study of CNS axon regeneration by enabling functional manipulation, molecular profiling, and three-dimensional structural visualization. These approaches have shifted the field from static, two-dimensional observations to dynamic, three-dimensional, and cell-type-specific analyses of axonal behavior. Future work integrating these technologies with advanced genetic tools (e.g., CRISPR screening) and longitudinal *in vivo* imaging will further elucidate the molecular logic underlying axonal structural plasticity and regeneration. Such insights are essential for designing targeted therapies that restore not only axonal connections but also meaningful functional recovery after CNS injury.
